# Efficient splicing of the CPE intein derived from directed evolution of the *Cryptococcus neoformans* PRP8 intein: Directed evolution of the *Cryptococcus neoformans* PRP8 intein

**DOI:** 10.3724/abbs.2023135

**Published:** 2023-07-23

**Authors:** Qin Zhan, Changhua Shi, Yu Jiang, Xianling Gao, Ying Lin

**Affiliations:** 1 College of Biological Science and Medical Engineering Donghua University Shanghai 201620 China; 2 Shandong Guoli Biotechnology Co. Ltd. Jinan 250101 China

**Keywords:** *Cne* PRP8 intein, C-extein, directed evolution

## Abstract

Intein-mediated protein splicing has been widely used in protein engineering; however, the splicing efficiency and extein specificity usually limit its further application. Thus, there is a demand for more general inteins that can overcome these limitations. Here, we study the *trans*-splicing of CPE intein obtained from the directed evolution of *Cne* PRP8, which shows that its splicing rate is ~29- *fold* higher than that of the wild-type. When the +1 residue of C-extein is changed to cysteine, CPE also shows high splicing activity. Faster association and higher affinity may contribute to the high splicing rate compared with wild-type intein. These findings have important implications for the future engineering of inteins and provide clues for fundamental studies of protein structure and folding.

## Introduction

Inteins are internal protein segments that can self-excise from their host protein and ligate the flanking sequence (termed extein) with a native peptide bond to yield a mature protein. This process is called protein splicing [ 1, 2] . Until now, over 900 inteins have been found in all three domains of life, including eukaryotes, eubacteria and archaea [3]. The standard splicing mechanism is directed by the four conserved motifs (A, B, F, and G) of inteins plus the first residue of C-extein, which can be briefly divided into four steps. Step 1, an N-O/N-S acyl transfer converts the peptide bond connecting N-extein and intein to a thioester or ester, thereby forming an ester or thioester intermediate. Step 2, transesterification joins the N-extein to the side chain of the first residue of C-extein, yielding a branched protein intermediate. Step 3, the cleavage process is performed by cyclization of the last residue (Asn or Gln), which causes intein to be cleaved, and the two exteins are ligated by an ester bond. Step 4, the ester bond spontaneously and rapidly changes to an amide bond, and the cyclized asparagine converts to succinimide by hydrolysis [ 4– 7] . Protein splicing has been proven to be a highly valuable tool for many applications in the fields of biotechnology and biochemistry both *in vivo* and *in vitro*, such as site-specific protein labelling [ 8– 10] , protein ligation, and cyclization [ 11– 15] . Replacement of the conserved residues of an intein will lead to N- or C-terminal cleavage reactions, which are widely used in protein purification [ 12, 16, 17] .

Although inteins have been successfully used in many cases, their applications are usually plagued by extein specificity and splicing efficiency. Sometimes inteins are inactive or inefficient when inserted into heterogeneous proteins [ 18, 19] . The extein sequences immediately flanking the intein affect splicing and can be defined as the intein substrate. *Npu* DnaE split intein (Npun_F4872, UniProtKB B2J066; Npun_F5684, UniProtKB B2J821) is one of the most efficient split inteins with reported rates of protein *trans*-splicing [(1.1 ±0.2)×10 ^–2^  s ^–1^] and high splicing efficiency [20]. It shows more sequence tolerance at the extein-intein junctions and is used for the efficient production of native disulfide-rich backbone cyclic polypeptides such as cyclotides, trypsin inhibitor SFTI-1, several θ-defensins, and a genetically encoded RTD-1-based peptide library in different heterologous expression systems [ 14, 21– 24] . However, mutation of the first (+1) C-extein residue from cysteine to serine in the host protein dramatically reduced the splicing efficiency; even by 24 h, the splicing efficiency was less than 30% [ 25– 28] . All these factors will limit their applications in biotechnology and chemical biology. Thus, it is important to identify more general inteins to solve these problems.

The *Cne* PRP8 intein (PRP8, UniProtKB Q58TX5) from *Cryptococcus neoformans*, an important fungal pathogen of humans, is the second intein found in a eukaryote nuclear genome. In a previous study, the wild-type *Cne* PRP8 mini-intein was shown to have robust protein splicing in *Escherichia coli* [29]. Then, it was subjected to six rounds of sequential directed evolution at the 138Ser insertion site in the kanamycin resistance protein (KanR). One mutant intein (CPE) was selected with significantly improved splicing efficiency [30]. In this study, the evolved CPE intein was further characterized and showed strong versatility and attractive *trans*-splicing kinetics [ *Kobs*=(9.0 ±0.1)×10 ^–3^  s ^–1^], which was expected to be a more widely used tool in the fields of biotechnology and biochemistry.

## Materials and Methods

### Plasmid construction

The *Cne* PRP8 intein was amplified by PCR using the primer set CPb1 and CPb3 from pMCP, which contains the full-length intein sequence [31], and then digested with *Bsa*I inserted into the pKH vector derived from pDrive (Qiagen, Hilden, Germany) by adding a 6×His tag before KanR to obtain pKCP138S (138 indicated the site of serine in KanR).

Plasmids pKCP (Q114R), pKCP (T133I) and pKCP (N139D) were derived from previously directed evolution in our laboratory. Plasmids pKCP (T52I), pKCP (S68P), pKCP (S159R) and pKCP (G122C*) were constructed by site-directed mutation of CPE with primers T52I-1/2, S68 P-1/2, G122C*-1/2 and S159R-1/2, respectively ( Supplementary Table S1).

As mentioned previously, plasmids for studying *cis*- or *trans*-splicing in the pMST system were constructed [ 31, 32] . The intein segments were amplified by PCR using the primers CPm1 and CPm2, digested with *Xh*oI and *Age*I, and ligated into the pMST plasmids with the same enzyme digestion. A spacer sequence (5′-TAGTTAAGGAGGAAAAACATATG-3′) consisting of a stop codon, a ribosome-binding site, and a start codon was inserted into the 129th amino acid of the intein segments by inverse PCR, resulting in pMCPS0 and pMCPES0 (S0 represented the split site). The CPE S0 split intein coding gene contains a two-gene operon, where the first gene encodes the N-protein consisting of a maltose-binding protein (M) and N-intein, while the second gene encodes the C-protein consisting of the C-intein and thioredoxin (T).

For *in vitro trans*-splicing, plasmids pMCPS0 and pMCPES0 were digested by *Nde*I and *Pst*I to obtain the C-protein gene and then ligated into pTWIN with the same enzyme digestion, resulting in pECPS0C and pECPES0C for expression of the C-protein, and the plasmids for N-protein expression, pMCPS0N and pMCPES0N, were obtained by blunting the cohesive ends and ligation. The plasmids for the study of surface plasmon resonance (SPR) and mutation analysis were created by directed site mutation using the primers CP0Na-1/2 and CP0Ca-1/2 ( Supplementary Table S1).

### Directed evolution of *Cne* PRP8 intein

The mutant pool was created from pMCP by error-prone PCR with the primers CPb1 and CPb3. Three groups of different error-prone PCRs were performed to obtain high, medium and low mutation rates [32]. These mutants were digested by *Bsa*I and purified, ligated with the evolution vector pKH and transformed into *E*. *coli* for screening. The recombinant plasmids were transformed into *E*. *coli* and plated on 100 μg/mL ampicillin to quantify the library size, as well as plates with 10, 20, 30, 50, 60 and 70 μg/mL kanamycin for each round of selection, incubated at 37°C for 12–16 h. To evaluate the ability of the insertion sites for directed evolution, we calculated the ratios of clones from plates with ampicillin and kanamycin. The templates of the next rounds were from the last rounds, and the first-round template was directly from pMCP.

### Protein expression and purification

For protein expression, each expression plasmid was transformed into *E*. *coli* BL21 (DE3) using standard transformation methods. Subsequently, the cells were grown at 37°C in liquid LB medium containing ampicillin to mid-log phase (OD _600_≈0.5), and IPTG was then added to induce protein expression at 25°C overnight. The overnight-induced cells were collected and lysed in hot SDS-PAGE sample buffer.

The N-proteins and C-proteins for studying *trans*-splicing *in vitro* were purified using amylose resin (New England Biolabs, Ipswich, USA) and Ni-NTA resin (Qiagen), respectively, according to the manufacturers’ protocols.

### Affinity study of the two halves of intein

The SPR study was performed on a Biacore T100 (GE healthcare, Bethesda, USA). The N-proteins (WTNa and ENa) were immobilized on the Biacore CM5 chip according to the manufacturer’s instructions. The C-terminal fusion proteins were diluted to different concentrations in running buffer (10 mM HEPES, 150 mM NaCl, 1 mM EDTA, pH 7.4) for the kinetics assay [ 31, 33] . Biacore T100 evaluation software was used to calculate the dissociation constant ( *kd*), association constant ( *ka*), and affinity constant ( *K _D_*). The equilibrium dissociation constant is *K _D_* = *kd*/ *ka*.

### Analysis of the splicing reaction

The collected protein samples were subjected to polyacrylamide gel electrophoresis (SDS-PAGE) or western blotting detection using anti-His/Thioredoxin/Maltose binding protein antibodies. In the KanR screening system previously constructed in directed evolution, both the N-terminus of the *KanR* resistance gene and the CPE intein have a 6× His-Tag, which were used for western blotting detection and analysis of the effect of different mutation sites on splicing efficiency. Splicing efficiencies in SDS-PAGE or western blotting were calculated from densitometry analysis of protein bands using ImageJ software.

## Results

### *Trans*-splicing activity and versatility of the CPE intein

**Figure 1:**
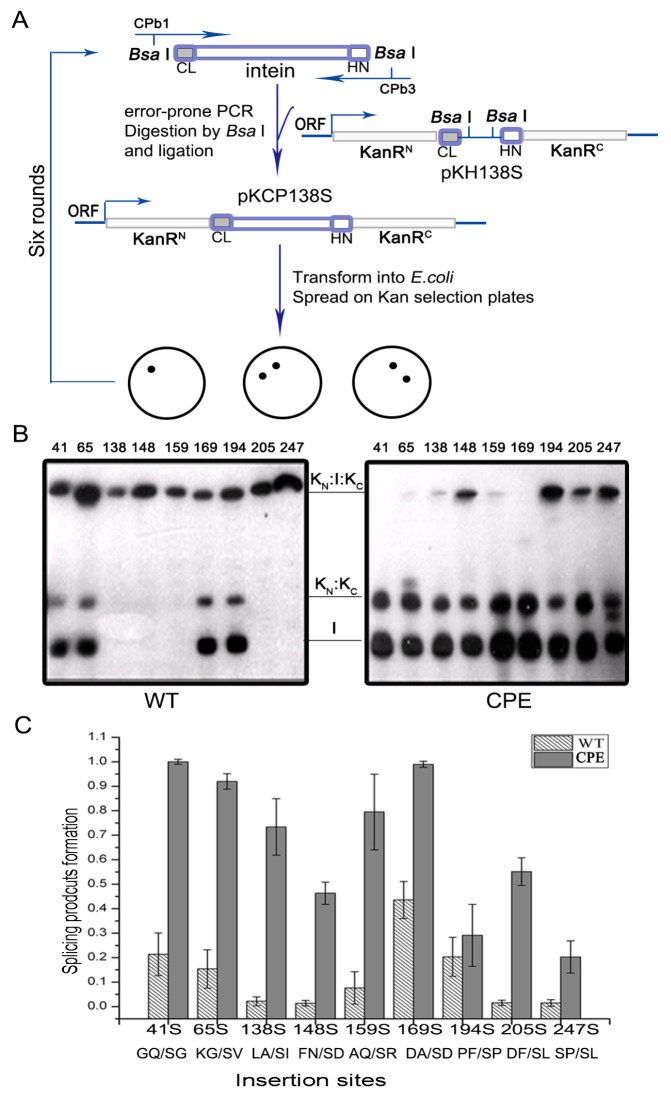
Figure 1 Directed evolution of the *Cne* PRP8 intein (A) Schematic of directed evolution to select an evolved intein. The intein mutants were generated by error-prone PCR and then inserted into a KanR protein, and the transformants containing the mutants were screened from LB plates with kanamycin. Six rounds of screening were performed to obtain the final evolved intein (CPE). (B) The splicing efficiency in all the serines in the KanR protein of wild-type intein (left) and the mutant intein (right) by western blotting using anti-His antibody. The insertion sites are indicated in the figure. (C) Comparing the splicing efficiency of wild-type (WT) and the evolved intein (CPE) in KanR before serine. The insertion sites and flanking residues are indicated.

A previously reported *Cne* PRP8 mini-intein was chosen for directed evolution. In a previous study, this intein was shown to splice efficiently (100%) in a foreign protein context (pMST) with five native neighboring extein residues [31]. However, when this intein was inserted in the KanR protein without the five native extein residues, it was unable to splice. The wild-type *Cne* PRP8 mini-intein was then subjected to six rounds of directed evolution at the 138Ser insertion site in the KanR protein. One mutant intein (CPE) that contained seven mutations (T52I, S68P, Q114R, T133I, N139D, S159R, G122C*) was selected with significantly improved splicing efficiencies ( Figure 1).

**Figure 2:**
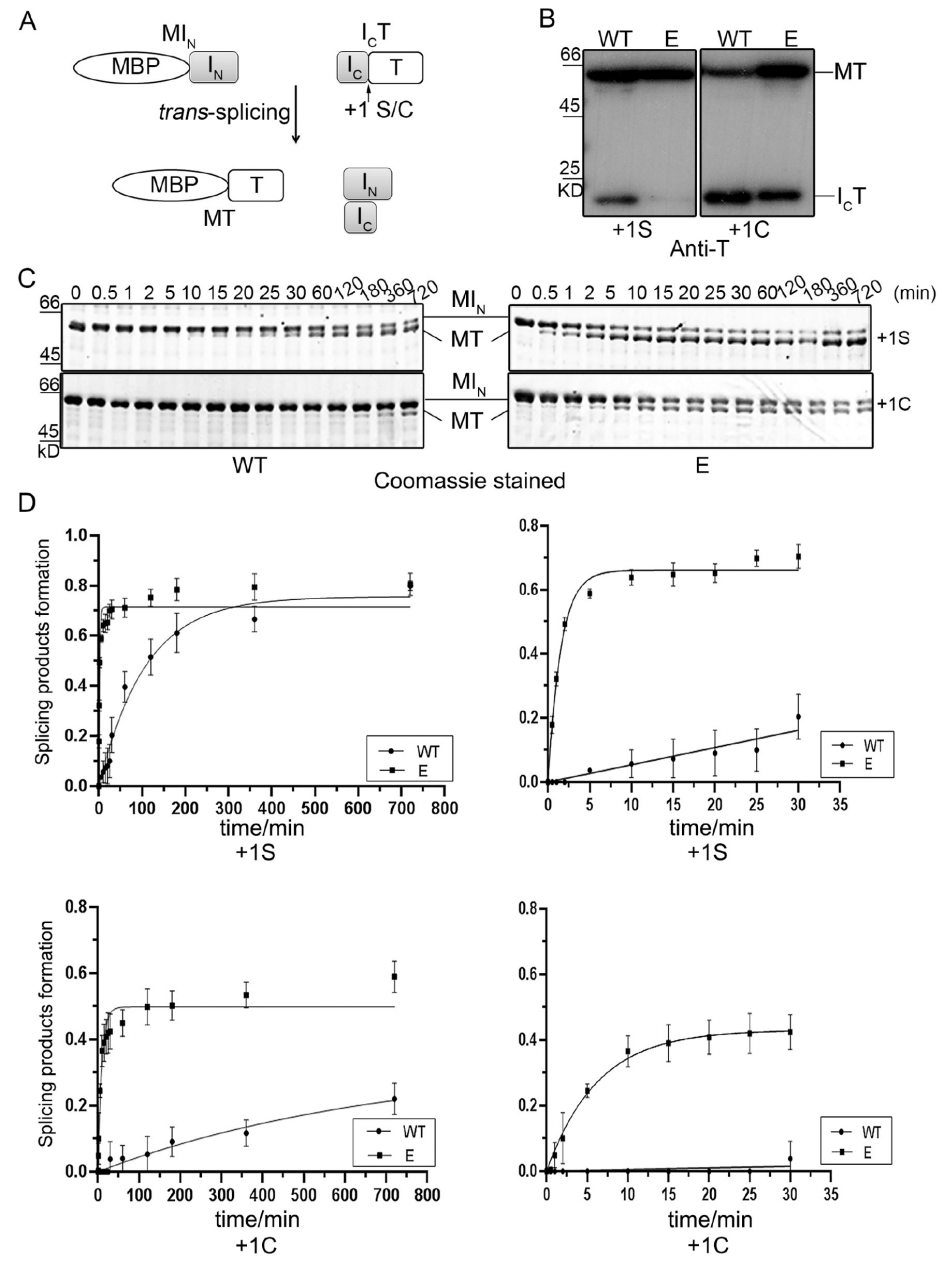
Figure 2 Comparison of the splicing efficiency of WT and CPE intein in the MST system (A) Scheme of the pMST system. (B) Identification of splicing products by western blotting analysis using anti-thioredoxin for in vitro protein splicing. (C,D) Comparing the trans-splicing kinetics of wild-type and CPE in vitro when the +1 residue of the extein is serine (S) or cysteine (C). All experiments were performed in triplicate, and error bars represent standard deviation. For the in vitro study, the N-protein and C-protein precursors were purified by amylose resin and Ni-NTA, respectively. The purified C-proteins (I CT) were mixed in threefold molar excess with N-protein (MI N), and DTT was added to a final concentration of 1 mM. At several time points, aliquots of the reaction mixtures were removed, and SDS-PAGE loading buffer was added to stop the reaction. The reaction products were resolved by SDS-PAGE and visualized by Coomassie blue staining.

To further study the *trans*-splicing activity, inteins were introduced into the maltose-binding protein-intein-thioredoxin (MST) system ( Figure 2A). The wild-type (WT) and the evolved intein (CPE) were cloned into the pMST vector with five native extein amino acids using maltose-binding protein (M) as the N-extein and thioredoxin (T) as the C-extein [31]. In *E*. *coli*, both the mini-intein and S0 split intein could gain almost 100% splicing activity ( Supplementary Figure S1). For a more quantitative analysis, the *trans*-splicing rates (k) of S0 intein from the time course experiments were determined. The purified N-protein (MI _N_, 57.3 kDa) and C-protein (I _C_T, 19.9 kDa) were mixed at a molar ratio of 3:1, and DTT was added to a final concentration of 1 mM. The nucleophilic residue at the +1 position immediately after intein is required for splicing. The CPE intein was also shown to perform better than the wild-type before different C-extein residues (+1Ser or +1Cys) ( Figure 2B).

To investigate the reaction kinetics of CPE intein, several time points were selected, and an aliquot of the reaction mixture was removed. Then, SDS-PAGE loading buffer was added and heated to stop the reaction. The amounts of the splicing products were calculated as a percentage of the initial amounts of N-protein. The splicing products (MT, 56 kD) can be detected on both gels, and there were no cleavage products detected in the process. Interestingly, the evolved intein has an unusually high *trans*-splicing rate ( Figure 2C,D). For a better understanding of the kinetics and more clarity, the reaction in the first 30 min is shown alone ( Figure 2D, right panel). According to the pseudofirst-order reaction equation of P=P _0_ (1–e- *^kt^*) [34], the splicing rate constant of the mutant intein ( *k _CPE_*) was determined to be 9.0 ±0.1×10 ^–3^  s ^–1^, which is ~29- *fold* higher than that of the wild-type [ *k _WT_* =(3.1 ±0.1)×10 ^–4^  s ^–1^], and most of the splicing occurred by 360 min. The high splicing rate of the evolved mutants was much more obvious when +1Ser was mutated to +1Cys, the evolved intein was ~75- *fold* higher than the wild-type [(1.8±0.1)×10 ^–3^ *vs* (2.4±0.1)×10 ^–5^], and almost all splicing took place by 60 min.

### The recognition of two halves of intein

Next, we explored the mechanism of the relatively high splicing rate of the evolved intein. Regarding the high splicing rate, it was supposed that the evolved intein may have a high speed of recognition and formation between the two halves to be the active state. In an effort to gain insight into the hypothesis, the rate of formation was measured by surface plasmon resonance. To prevent splicing and cleavage during this process, we introduced C1A (the first residue of intein) and N185A (the last residue of intein) in N-proteins and C-proteins, respectively, resulting in MI ^A^_N_ and I ^A^_C_T. The association rate constant ( *Ka*) of CPE was determined to be 3.199×10 ^4^ M ^–1^s ^–1^, which is ~6- *fold* higher than that of the wild-type (4.99×10 ^3^ M ^–1^s ^–1^), indicating that the evolved intein has a faster rate of formation of the two halves. As expected, the *K _D_* for CPE was determined to be 2.75×10 ^–7^ M, while the *K _D_* for the wild-type was 1.649×10 ^–6^ M ( Table 1, Supplementary Figure S2).

**Table:** **Table 1** Summary of the affinity kinetics of two halves of intein by SPR

Analyst	Ligand	*k _a_* (M ^–1^s ^–1^)	*k _d_* (s ^–1^)	*K _A_* (M ^–1^)	*K _D_* (M)
WTCa	WTNa	4.990×10 ^3^	8.2×10 ^–3^	6.08×10 ^5^	1.649×10 ^–6^
ECa	ENa	3.199×10 ^4^	8.7×10 ^–3^	3.67×10 ^6^	2.750×10 ^–7^

### Terminal cleavage assay

**Figure 3:**
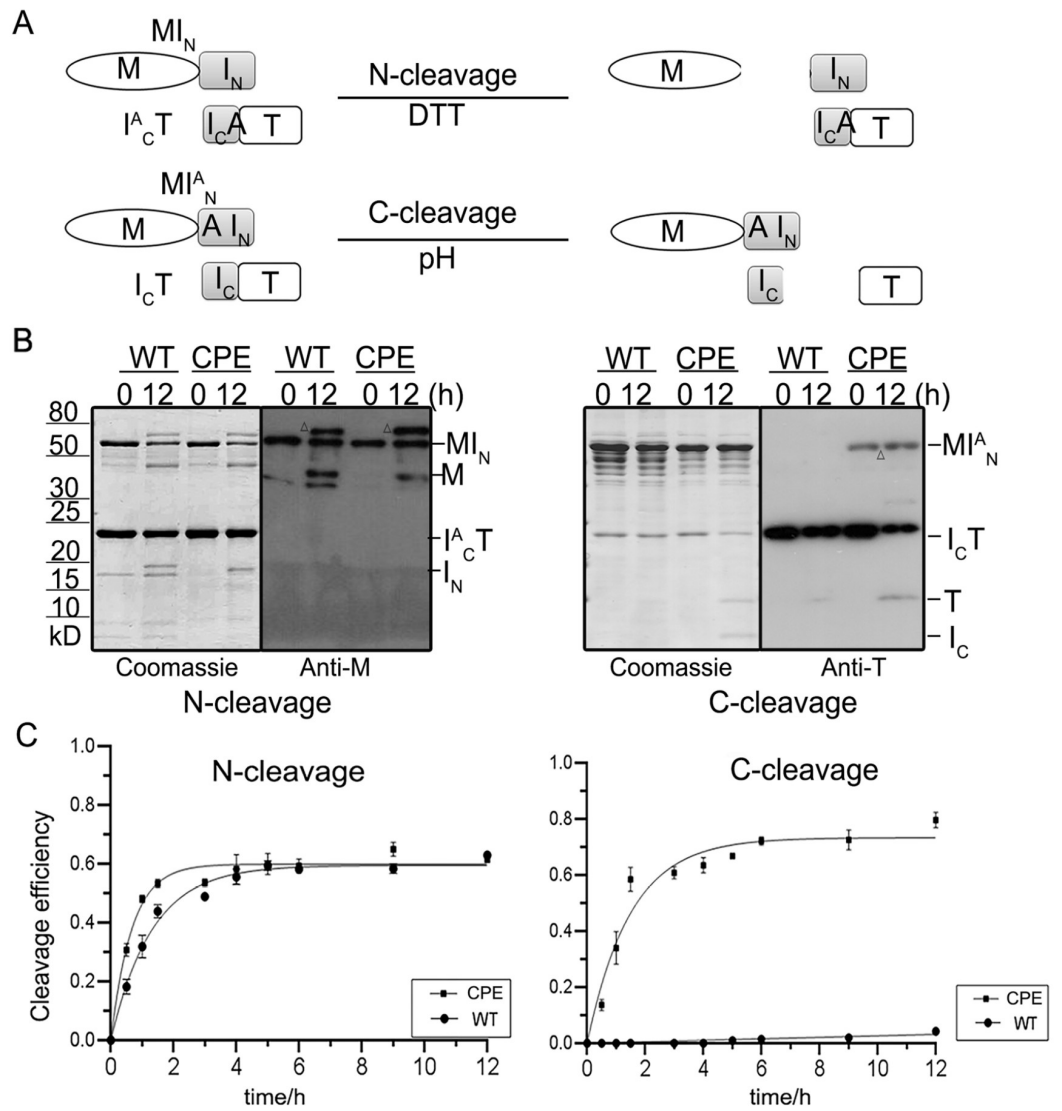
Figure 3 Terminal-cleaving reactions of WT and CPE intein in the MST system (A) Overview of the terminal-cleaving reactions. (B) The cleavage products were either stained with Coomassie blue or probed with the indicated primary antibody. MI N and I ACT are precursors of in vitro N-terminal cleavage reactions, while M and I N are cleavage products. MI AN, I CT, and T, I C are precursors and products of the C-terminal cleavage reaction, respectively (right). △, unspecific bands. (C) Time course of the formation of cleavage products at the N-terminus and C-terminus of the wild-type (WT) and evolved (CPE) inteins.

To fully characterize the evolved CPE intein, the kinetics of the terminal cleavage reaction were analyzed. Usually, intein *trans*-splicing is a five-step reaction, including the recognition of the two segments, N-S (or N-O) acyl shift, transesterification, Asn cyclization and uncatalysed S-N (or O-N) acyl shift. The linear and branched (thio)ester intermediates are prone to cleavage by hydrolysis or attack by other nucleophiles, leading to cleavage of the N-terminal scissile bond, referred to as N-cleavage. Premature succinimide formation leads to C-cleavage. The *trans*-splicing intein fragments can be converted into *trans*-cleaving elements by substituting some key residues in the splicing reaction [ 7, 35] . Most inteins are able to perform C-cleavage in the presence of a C1A mutation. To achieve N-cleavage, the intein’s last residue is mutated to abolish the Asn cyclization and S-N (or O-N) acyl shift of the splicing mechanism. The N-S (or N-O) acyl shift with/without transesterification can still occur, and the resulting ester bond can spontaneously hydrolyse to separate the N-extein from the intein. Here, we applied MI ^A^_N_ to induce the C-terminal cleavage of I _C_T and I ^A^_C_T to induce the N-terminal cleavage of MI _N_, by pH shift and DTT, respectively ( Figure 3A). After 12 h of incubation, the cleaving products (M and I _N_) can be detected both from the wild-type and the evolved inteins. However, an unexpected cleaving product that was higher than MI _N_ appeared on the gel and western blotting film ( Figure 3B left). By comparing the size of the bands with the noncleaving mutant (MI ^AA^T), MI _N_T, MI _C_T, and MT, we found that this unexpected band probably belongs to MI _C_T (data not shown). The final cleaving efficiency was calculated by the density of the I _N_ fragment on the gel, and the N-terminal cleaving rates of the WT and CPE were very close (WT 1.55×10 ^–4^  s ^–1^ *vs* 6.58 ×10 ^–4^  s ^–1^; Figure 3C left).

In the C-terminal cleavage reaction, the advantages of the evolved intein are much more obvious than those of N-terminal cleavage. After 12 h of incubation, the cleaving efficiency of the wild-type is lower than 10%, while the efficiency of evolved intein can reach 60% in 2 h of incubation, and it remains stable by 6 h. The fitting cleaving rate is also faster than the existing C-terminal full-length intein rate ( *k*=1.47×10 ^–4^  s ^–1^; Figure 3B,C right). This showed that CPE mainly accelerated the penultimate step of the splicing reaction process.

### The roles of the mutations in CPE intein

**Figure 4:**
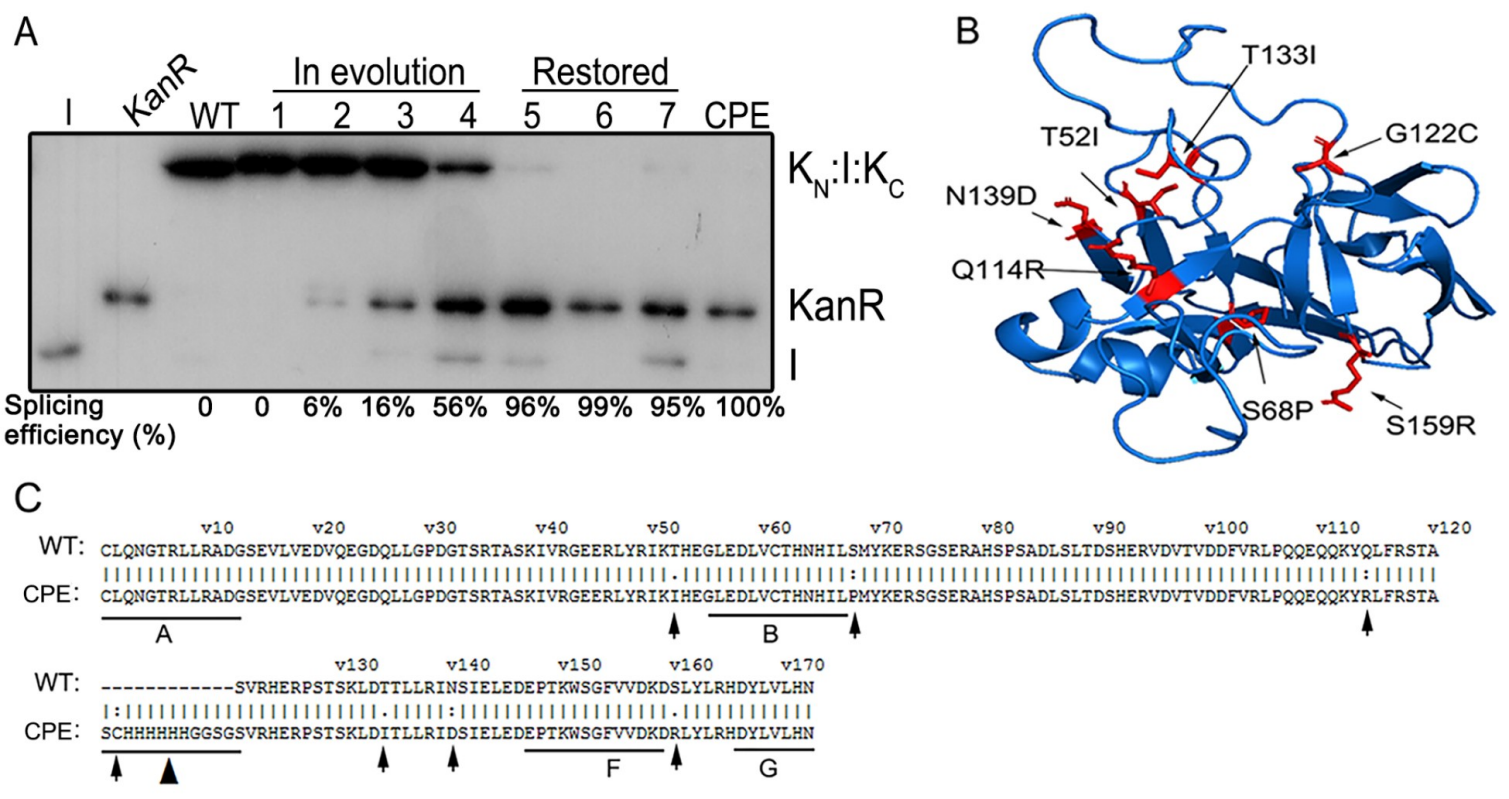
Figure 4 Analysis of the roles of the mutation in the evolved intein (A) Western blotting analysis was used to detect the splicing efficiency of different mutants. 1‒4 are the single mutant (T52I), double mutant (T52I and T133I), three-point mutant (T52I, T133I, and N139D) and four-point mutant (T52I, T133I, N139D, and Q114R). 5‒7 are mutants that restore the amino acid positions of S68P, G122C*, and S159R, respectively. All mutations are relative to the wild-type sequence of the Cne PRP8 mini-intein. (B) Structural modelling of the CPE mini-intein. Computer-based modelling of the Cne PRP8 intein was performed using the SWISS-MODEL program (https://swissmodel.expasy.org/) based on the crystal structure of the Cne PRP8 mini-intein [41]. Mutated residues in the intein are shown in red. (C) Comparison of amino acids of the wild-type (WT) and the evolved intein (CPE). Mutated residues are indicated by arrows in B and C. The sequence marked by ▲ is an artificial linker (SGHHHHHHGGSG), and mutation G122C* is in this linker where the putative endonuclease domain inserts.

To study the roles of mutations in the splicing and cleavage efficiency of CPE inteins, we briefly analysed them. There are six mutations in the CPE intein, except one (G122C*) in an artificial linker that replaces the endonuclease domain ( Figure 4B,C). During the previous evolutionary process, we found that four mutations (T52I, T133I, N139D, Q114R) commonly occurred and accumulated well in the six rounds of evolution ( Supplementary Table S2). To study the roles of these four mutants, expression plasmids containing double mutations (T52I and T133I), three-point mutations (T52I, T133I, and N139D) or four-point mutations (T52I, T133I, N139D, and Q114R) were directly obtained in evolution, while the single mutation (T52I) was constructed by site-directed mutagenesis. A single mutation (T52I) showed less than 5% splicing, while mutations (T52I and T133I) showed 6% splicing. When the mutations were increased to three (T52I, T133I, and N139D) and four (T52I, T133I, N139D, and Q114R), splicing efficiency was increased to 16% and 56%, respectively ( Figure 4A).

The roles of the other three mutations were determined by restoring the mutation and comparing the splicing efficiency to the CPE intein. The results showed that in the absence of the S68P and S159R mutations, the final splicing was reduced by 1%~10%. The role of the G122C* mutation was not as obvious as that of the previous two mutations ( Figure 4A).

## Discussion

In this study, the CPE intein derived from previously directed evolution in our laboratory showed a more general ability to efficiently splice in non-natural extein sequences. To our surprise, when the +1 residue of the extein is serine, the *trans-*splicing rate of the intein is increased by approximately 29- *fold* compared to the wild-type, and when the +1 residue of the extein is replaced by cysteine, its *trans*-splicing rate is increased by approximately 75- *fold* ( Figure 2). Through SPR, we detected the process of the formation of the intein’s two halves in quantity and found that the mutant intein has a faster association rate and *K _D_* is smaller than the wild-type. The high formation rate could account for the high splicing rate of the mutant intein. Shah *et al*. [36] reported that split intein fragments assembled through the “capture and collapse” mechanism using *Npu* DnaE as the model intein. They showed that the interaction occurred through a multiphase process initiated by electrostatic interactions between extended regions of both fragments. We also calculated the pI values for both the wild-type and evolved CPE intein fragments (S0N/S0C: pI=5.97/6.51 *vs* pI=5.5/6.51), which were not significantly different. They also proved that introducing repulsive electrostatic interactions at the fragment interface diminished splicing activity and that compensatory electrostatic mutations recovered splicing activity. This effect on activity is correlated with the binding affinity. Therefore, mutations at the fragment interface may contribute to the binding affinity and relatively high splicing rate of CPE intein.

Among all reported fast-splicing inteins, such as *Npu* DnaE [20], GP41-1 [37], and *Cfa* DnaE [38], the *trans*-splicing rate of CPE intein is almost equivalent to the best characterized naturally split intein *Npu* DnaE. Compared with *Npu* DnaE, it splices at a higher rate before both serine and cysteine residues. When *Npu* DnaE is placed before serine, splicing efficiency and splicing rate are significantly reduced. Although previous work from Cheriyan *et al*. [25] showed a mutant *Npu* DnaE that can undergo splicing before a serine with relatively higher efficiency, it needs more than 20 h to complete the reaction. Based on this, CPE inteins may be more widely used for the semisynthesis of proteins.

However, when the terminal cleavage efficiency of the CPE intein was measured, its N-terminal cleavage had little change compared to the wild-type, which may be because its wild-type efficiency is already particularly high. The unexpected bands appeared during this process may be formed by the N-terminal cleavage product M being somehow linked to the precursor IcT or protein contaminants. At the same time, its C-terminal cleavage efficiency was increased by almost 6- *fold*, which is very encouraging ( Figure 3). This shows that CPE accelerates the splicing reaction in step 2. Previous studies on evolved *Mtu* RecA intein and the protein splicing or cleavage showed the mutants act synergistically to increase both splicing and C-terminal cleavage rates [39]. A recent report on *Ssp* DnaB and its M86 mutant showed a high degree of plasticity in the kinetic coordination of the splicing pathway for maximum product yield of ligated exteins [40].

The CPE intein contains six mutations in the protein splicing domain, except one (G122C*) in the artificial linker. The numbering of the mutation position in the CPE in this study is different from the previous report [30] in which the artificial linker was included, whereas the original position of the CPE intein was adopted in this article. All six mutations contribute to the final splicing efficiency to different degrees, and they are randomly located in the intein sequence, which makes it difficult to predict mutation sites. When CPE’s three mutation sites (S68P, S159R, and G122C*) were restored, there was a certain amount of deviation in the splicing efficiency compared with the previous report. Modelling of CPE was generated based on the crystal structure of *Cne* PRP8 ( Figure 4) [ 41, 42] . The residues of active sites consist of Cys1, Thr62, His65, His183, Asn184, and +1Ser, which provide hydroxyl groups and result in transesterification. Viewed against the whole structure, four mutations are located near the intein active site, and two are away from the whole horseshoe-like region. In detail, two of them (T52I and S68P) are relatively lose to active sites, two (Q114R and S159R) were further away from the active sites, and the other two (N139D and T133I) were located in the large loop ( Figure 4B). The hydrophilic threonine being mutated to hydrophobic isoleucine may lead to the residues’ side strains migrating toward the molecule core and affecting the direction of His65 by a hydrogen bond. The Q114R and S159R mutations provide more cationic residues. T133I mutation forms a large hydrophobic area by the side-chain atoms of Ile138, Leu135, Leu136, Leu143, and Ile142, and this conformation would be favorable for structure formation. T52I, S68P, Q114R and S159R are all in the predicted β-sheet of the intein structure.

Intein is usually inserted into a flexible region, which can guarantee the refolding of the intein and splicing activity. The inherent splicing ability of an intein is dependent upon the structure. Although the amino acid sequence of inteins is usually not highly homologous, most inteins have a horseshoe-like structure, and the two ends of the intein are located in the core of the structure and close to each other [19]. In the splicing mechanism, the flanking extein residues also need to be contained in the active core. If there is not enough reserved space, the exteins may not be embodied in the core, and splicing may not occur. In the present study, we found that CPE intein can be efficiently spliced in unnatural +1 extein (cysteine). We hypothesized that the evolved intein may have a more suitable active site that fulfils many types of extein residues to fold into the active site to process protein splicing. In the previous evolutionary system, compact insertion sites may partially contribute to the eventual universality of the mutant protein. To understand the relationship between the structure and function, we need to learn the structure of the intein in detail.

More general and efficient inteins will be important to increase the possibility of success by less limiting extein specificities. In conclusion, we analysed CPE intein and proved that it is more efficient and versatile in this study. We have reason to believe that the new mutant intein will expand the use of intein in all life science and chemical fields.

## Supporting information

Supplementary

## Supplementary Data

Supplementary data is available at *Acta Biochimica et Biphysica Sinica* online.
