# Supplementary material for: Efficient splicing of the CPE intein derived from directed evolution of the Cryptococcus neoformans PRP8 intein : Directed evolution of the Cryptococcus neoformans PRP8 intein
Source: Acta Biochim Biophys Sin (Shanghai). 2023 Jul 23;55(8):1310–8. doi: 10.3724/abbs.2023135 (PMC10448054; doi:10.3724/abbs.2023135)
Supplement: Supplementary [file Supplementary.pdf]

Supplementary Materials

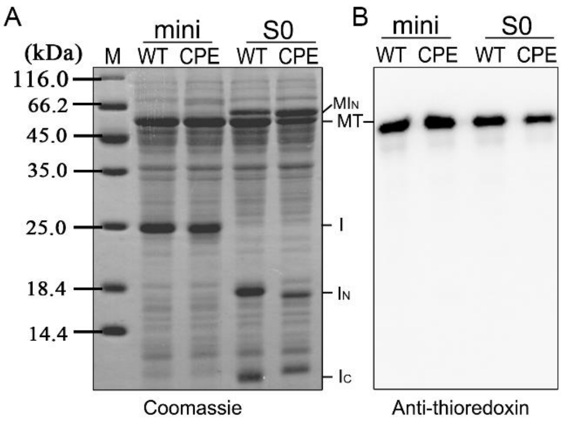

Supplementary Figure S1. Protein splicing of wild-type (WT) and mutant (CPE) intein in *E. coli* For mini-intein, the plasmids expressing precursor protein (MIT) containing a mini-intein were transformed into *E. coli* cells for expression. For S0 split intein, the N-protein (MI_N) and the C-protein (I_CT) were co-expressed in *E. coli* overnight at 25ºC. Total cellular proteins were resolved by SDS-PAGE. Protein bands were visualized by Coomassie blue staining (A) or by western blotting using an anti-T antibody as indicated (B).

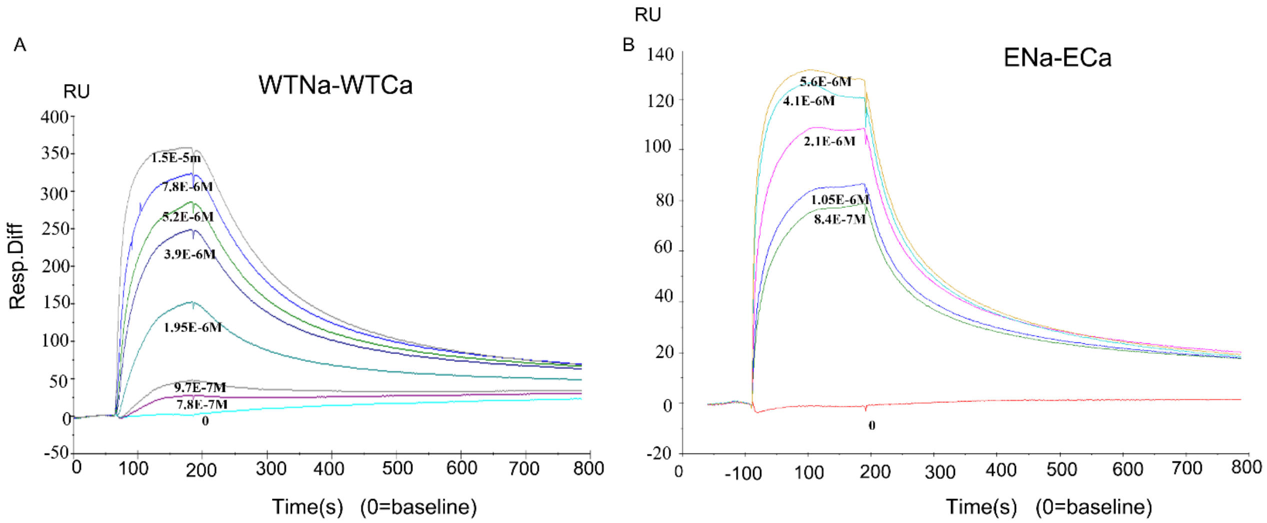

Supplementary Figure S2. SPR analysis of the S0 split inteins The SPR study was performed on Biacore T100. The N terminal fusion proteins were immobilized on Biacore CM5 chip according to the manufacture's instruction. (A) Wild-type S0 split intein. (B) CPE S0 split intein.

Supplementary Table S1. The sequences of primers used in this study

Primers	Sequence (5′→3′)
CPb1	ATCGGTCTCATGTCTTCAGAATACTCGT
CPb3	ATCGGTCTCAGTTGTGAATACCAAATAGTC
T52I-1	GATTTTGATACGGTAGAGACG
T52I-2	CATGAGGGGCTCAAGACCTT
S68P-1	CACATCCTTTCTATGTATAAAGAAAGGT
S68P-2	GTTATGGGTACAGACAAGATCTTC
G122C*-1	AGCGGTCATCACCATCATCACCATGG
G122C*-2	AGCAGTTGAACGGAAAAGCTGATACT
S159R-1	ACAGTCTTTATCTTCGTCATGACTA
S159R-2	CTTTGTCAACCACAAAACCGGACCACTTC
CP0Na-1	ACGCTCGAGTTTTGGGAGAAAGCCGCTCTTCAGAATGGGTACTCGT
CP0Na-2	CGATGTTCAGTTTTGCAACGGTCAGTT
CP0Ca-1	TCGATGAAGCCCTGAAAGACGCGCAG
CP0Ca-2	CATACCGGT CTCCTCAAAGCCTGAGGCGTGCAATACCAAATCGTCA

Supplementary Table S2. Mutation in the mutants of directed evolution

Mutants in the rounds of evolution	Mutations
The third rounds	Q3R, T52I, S68P, N139D
	T52I, S68P, T133I, H172R,
	T52I, T133I
	T52I, H127P
The fourth rounds	T52I, S81T, D100A, T133I, N139D
	T52I, T133I, N139D
	T52I, Q114R, T133I, N139D
The fifth rounds	T52I, D100E, Q114R, R117C, T133I, N139D
	T52I, Q114R, R117C, T133I, N139D
	T52I, L88R, Q108H, Q114R, S118G, T133I, N139D
	T52I, D101N, Q114R, R117C, T133I, N139D
The sixth rounds	T52I, S68P, Q114R, T133I, N139D, S159R, G122C*
